# Different Directions of Effects of Polyclonal IgG Antibodies from Patients with Schizophrenia and Healthy Individuals on Cell Death In Vitro: A Pilot Study

**DOI:** 10.3390/cimb45040206

**Published:** 2023-04-06

**Authors:** Elena V. Epimakhova, Liudmila P. Smirnova, Daria V. Kazantseva, Daria A. Kamaeva, Svetlana A. Ivanova

**Affiliations:** 1 Mental Health Research Institute, Tomsk National Research Medical Center of the Russian Academy of Sciences, Aleutskaya Str., 4, 634014 Tomsk, Russia; 2 Division of Biology and Genetics, Siberian State Medical University, Moskovsky Trakt, 2, 634050 Tomsk, Russia; 3 Department of Psychiatry, Addictology and Psychotherapy, Siberian State Medical University, Moskovsky Trakt, 2, 634050 Tomsk, Russia

**Keywords:** schizophrenia, abzyme, immunoglobulin G, SH-SY5Y cells, IgG catalase activity, IgG superoxide dismutase activity, cell death

## Abstract

Numerous studies indicate the involvemen of oxidative stress in the pathogenesis of schizophrenia. It has been shown that the serum pool of antibodies in patients with schizophrenia contains catalytically active antibodies (abzymes) that have a wide range of activities, including redox properties. In the present work, the effects of IgGs—having oxidoreductase activities—isolated from the serum of patients with schizophrenia and healthy individuals were studied in vitro. The IgGs were purified by affinity chromatography followed by an SDS-PAGE analysis of homogeneity in a 4–18% gradient gel. The catalase and superoxide dismutase (SOD) activities of the IgGs were measured spectrophotometrically using a kinetic module. Human neuroblastoma SH-SY5Y cells were cultured with IgG at a final concentration of 0.2 mg/mL for 24 h. In a parallel experiment, *tert*-butyl hydroperoxide was used as an oxidative stressor. The number of dead cells after incubation was determined with fluorescent dyes, propidium iodide and Hoechst, by high-throughput screening on the CellInsight CX7 platform. A cytotoxic effect of the IgG from the schizophrenia patients on SH-SY5Y cells was detected after 24 h incubation. A correlation was found between the SOD activity of the IgGs and IgG-induced cell death. Under the induced oxidative stress, the cytotoxic effect of the IgG from the patients with schizophrenia on the SH-SY5Y cell line was five times stronger. Meanwhile, the IgG from the healthy individuals exerted a cytoprotective effect on the cultured cells, accompanied by high catalase activity. Thus, the observed influence on cell viability depends on the catalytic properties of the abzymes.

## 1. Introduction

In recent years, it was found that immunoglobulins of various classes have an ability to not only bind an antigen but also to catalyze chemical reactions with it [1]. Such antibodies are called abzymes. Catalytically active antibodies were discovered in autoimmune diseases (e.g., Hashimoto’s thyroiditis, systemic lupus erythematosus, and multiple sclerosis) and later were also found in inflammatory diseases and in healthy people [2,3,4]. The presence of catalytic properties in antibodies substantially increases their functional potential but at the same time can lead to pathological effects; however, the biological role of abzymes in the pathogenesis of diseases remains poorly understood. Recently, schizophrenia was postulated as one of the diseases whose pathogenesis is associated with inflammation. In patients with schizophrenia, numerous studies have proved changes in the concentration of cytokines in the peripheral blood and cerebrospinal fluid, an increase in the concentration of neurospecific autoantibodies, and the activation of microglia [5,6,7]. Meanwhile, the functions of serum antibodies with catalytic properties have been under active investigation in recent years. Oxidative stress is also an important factor for the initiation and progression of this disease, as evidenced by an imbalance in the antioxidant defense system in patients during the first episode and patients with chronic forms of schizophrenia receiving long-term antipsychotic treatment [8,9,10]. Disturbances of the redox balance in schizophrenia lead to alterations in various metabolic and signaling pathways, thereby eventually contributing to aberrant neuronal development, hypomyelination, immune system dysfunction, and the development of inflammatory processes [11,12,13]. According to the literature, schizophrenia is associated with the hyperproduction of the superoxide anion owing to the activity of NADPH-dependent oxidase [14]. It is also known that oral haloperidol increases the formation of the superoxide anion in the body [12]. In addition, in patients with schizophrenia, the SOD activity in plasma and polymorphonuclear leukocytes is significantly reduced but is significantly increased in serum as compared to healthy individuals [15]. Studies have also shown a link between schizophrenia and polymorphisms of manganese superoxide dismutase (Mn-SOD) synthesis genes: a comparative proteomic analysis of postmortem human hippocampal tissue samples revealed that the concentration of Mn-SOD is significantly diminished in the hippocampus of a schizophrenia patient [16].

IgG SOD activity has been found in schizophrenia and multiple sclerosis, but the function of abzymes having antioxidant activity is not fully understood. Over the past few years, abzymes with nuclease [17,18], proteolytic [19,20], catalase [21], and superoxide dismutase (SOD) activities [22] have been found in the blood serum of patients with schizophrenia. One hypothesis about the pathogenesis suggests that catalytic antibodies with catalase and SOD activities help protect the body from excessive concentrations of reactive oxygen species (ROS) [23,24]. On the other hand, there is a hypothesis that catalytic antibodies have cytotoxic properties (they affect intracellular processes and activate membrane damage and apoptosis), which are especially pronounced in autoimmune diseases. For example, in systemic lupus erythematosus, polyclonal DNA-hydrolyzing antibodies have been found that influence the viability of cell lines L929, HL-60, Raji, and K562 [25]. It has been demonstrated that the cytotoxicity of such antibodies is predicated, first, on their ability to penetrate the cell by endocytosis and to cause nuclear DNA fragmentation, which leads to caspase-dependent cell death. Second, cytotoxic antibodies can interact with cell surface receptors, triggering apoptosis. In this regard, an in vitro experiment concerning the impact of antibodies having oxidoreductase activities from schizophrenia patients on cell death in a neuroblastoma cell model will allow us to establish the protective or cytotoxic properties of such IgGs.

## 2. Materials and Methods

### 2.1. Description of Participants of the Study

The investigation of catalase and SOD activities belonging to antibodies was performed in 16 patients with schizophrenia (the male/female ratio was 62.0%/38.0%). All patients were recruited from the Department of Endogenous Disorders at the Mental Health Research Institute of TNMRC (Tomsk, Russia). The median age of the patients was 40.50 years [interquartile range: 38.00; 45.50] with a minimum of 20 years and a maximum of 60 years. The diagnosis of paranoid or simple schizophrenia was made in accordance with the International Statistical Classification of Diseases and Related Health Problems, 10th Revision (ICD-10) and the Structured Clinical Interview for DSM-IV Axis I Disorders (SCID). The list of exclusion criteria was as follows: detection of an acute infectious, decompensated chronic disease or a site of inflammation; autoimmune or neurological pathologies; detection of organic or other mental disorders not included in the study; and mental retardation.

The control group was composed of 18 mentally and somatically healthy people (the male/female ratio was 39%/61%). The age of the participants in this group ranged from 21 to 65 years, and the median was 44.00 [35.00; 49.00] years. The exclusion criteria for the control group were the presence of an acute infectious, decompensated chronic disease or a site of inflammation, autoimmune or neurological pathologies, organic brain lesions, and the presence of mental disorders (Table 1).

The study protocol was designed according to the Helsinki ethics committee guidelines and was approved by the Local Bioethics Committee of the Mental Health Research Institute (protocol No. 157/1.2022). Written informed consent was obtained from each study participant before the study. Consent from next of kin was not required because the participants’ ability to give consent was not compromised.

### 2.2. Materials

Biomaterial preparation included blood sampling from the cubital vein into Vacutainer tubes (BD, Franklin Lakes, NJ, USA) containing a clot activator after an overnight fast. Blood samples were collected from the patients before the time of day when an antipsychotic drug was taken. Serum separation was performed by centrifugation at 2000× *g* and 4 °C for 20 min in a refrigerated centrifuge (Orto Alresa Digicen 21R; Madrid, Spain). Serum was aliquoted and stored at −80 °C until subsequent isolation of immunoglobulins.

### 2.3. Isolation of Serum Immunoglobulins on Protein G Sepharose

Immunoglobulins G were isolated from individual serum samples of schizophrenia patients and healthy controls by affinity chromatography on an ÄKTA Pure 25 chromatograph (GE Healthcare Bio-Sciences, Uppsala, Sweden). Serum after 3-fold dilution with Tris-buffered saline (TBS; 0.05 M Tris-HCl pH 7.5 and 0.15 M NaCl) was applied to a Protein G Sepharose column (HiTrap™ Protein G HP, GE Healthcare). After the sample loading, the column was washed to zero optical density at 280 nm (A_280_) with TBS. At the first step, the elution of lipids and nonspecifically adsorbed proteins was performed with TBS-Tween buffer (1% Triton X-100, 0.05 M Tris-HCl pH 7.5, and 0.3 M NaCl). IgGs were eluted with 0.1 M glycine-HCl buffer (pH 2.6). The eluate was fractionated and neutralized immediately with 1 M Tris-HCl buffer (pH 8.8). Between the stages of antibody purification, the columns were washed with TBS until A_280_ of the eluate reached zero [20]. Then, the homogeneity of IgGs was tested by SDS-PAGE analysis in a 4–18% gradient gel.

#### 2.3.1. Dialysis of Purified Antibodies

To remove low-molecular-weight substances, the isolated IgG antibodies were dialyzed. Immediately before dialysis, the dialysis membranes were boiled for 10 min to promote pore opening. The fractions of eluted antibodies were dialyzed against 0.02 M sodium phosphate buffer (pH 7.0) at 4 °C on a magnetic stirrer for 17 h [23].

#### 2.3.2. Protein Concentration Assessment

The IgG concentration was analyzed on a Varioskan LUX spectrophotometer (Thermo Scientific, Waltham, MA, USA) located at the core facility Medical Genomics at Tomsk National Research Center. IgG concentrations in the samples were calculated from a standard calibration curve for IgG.

### 2.4. Assessment of IgG’s Catalase Activity

IgG catalase activity was assessed by means of a decrease in absorbance caused by the degradation of hydrogen peroxide in accordance with ref. [26]. The reaction mixture consisted of 50 mM potassium phosphate (pH 7.0), 30 μM H_2_O_2_, and purified antibodies at 0.5 mg/mL. Spectrophotometric analysis was carried out at 240 nm within 5–8 min using a Cary 60 spectrophotometer (Agilent Technologies, Santa Clara, CA, USA). For the subsequent activity calculations, we chose only data from the time intervals showing a linear dependence of relative enzymatic activity on IgG concentration. The molar extinction coefficient of hydrogen peroxide for the calculation of the activity was taken from [21]. Specific activity was expressed in µM H_2_O_2_/min/mg IgG.

### 2.5. Assessment of IgG’s SOD Activity

The assessment of IgG SOD activity was based on the inhibition of the reduction of nitroblue tetrazolium by superoxide radicals. Oxidation of xanthine to uric acid in the presence of xanthine oxidase leads to the formation of superoxide radicals, which in turn results in reduced diformazan derived from nitroblue tetrazolium. The difference in the amount of reduced diformazan between samples without IgG (a spontaneous reaction) and samples manifesting the inhibitory effect of IgG SOD activity (an induced reaction) at a wavelength of 560 nm was employed to evaluate the enzymatic activity.

SOD activity of IgG was expressed in the following units: the difference in optical density between the spontaneous and induced reaction proceeding for 1 min in terms of mg of IgG (µM diformazan/min/mg IgG) [22].

### 2.6. Assessment of Cell Viability

The influence of IgG samples on cell viability was evaluated on cultured human neuroblastoma SH-SY5Y cells. SH-SY5Y cells were cultured at 37 °C, 90% relative humidity, in a humidified atmosphere containing 5% of CO_2_ (in a CO_2_ incubator: Sanyo MCO-5AC, Japan) in the DMEM medium supplemented with 50 U/mL penicillin, 50 µg/mL streptomycin, and 10% of fetal bovine serum. This culture medium was refreshed every 2–3 days.

To investigate the effects of IgG on cell viability, SH-SY5Y cells were seeded in 96-well plates (10^4^ cells per well), and a day later, IgG antibodies were added at a final concentration of 0.2 mg/mL, followed by culturing for 24 h. As a control culture, we utilized (1) SH-SY5Y cells grown in the aforementioned culture medium without the addition of IgG and (2) B culture, in which an equivalent volume of phosphate buffer pH 7.0 (the buffer for antibody dialysis) was added instead of IgG. The indicated concentrations of IgG antibodies was selected empirically in previous experiments [27].

To assess properties of the immunoglobulins under oxidative stress, *tert*-butyl hydroperoxide (TBH) was added to SH-SY5Y cells at a final concentration of 500 μM. SH-SY5Y cells were incubated with IgG as described above (0.2 mg/mL IgG for 24 h), and then TBH was added for 4 h incubation at the above concentration. The indicated concentration of TBH was chosen according to results of our own experiments [28].

Dead cells were counted on the CellInsight CX7 HCS platform (Thermo Fisher Scientific, USA) at the core facility Medical Genomics (Tomsk Scientific Research Center) by using fluorescent dyes Hoechst (stains all cell nuclei, signal registration at 386 nm) and propidium iodide (stains the nuclei of dead cells, signal registration at 560 nm). Nuclei were then identified and analyzed based on signal fluorescence intensity in HCS Studio™ cell analysis software (Thermo Fisher Scientific).

### 2.7. Statistics

Statistical analyses were performed in SPSS, version 23 (IBM Company, Westchester, NY, USA). The data were tested for the normality of the distribution by the Shapiro–Wilk test. Description statistics are shown as a median with 25% and 75% quartiles (Me [Q1; Q3]) or mean and standard deviation (m ± SD). Differences were considered statistically significant at *p*-values less than 0.05. Between-group differences were evaluated by Student’s *t* test for independent samples with a normal distribution.

## 3. Results

### 3.1. Application of Stringent Criteria to Define Oxidoreductase Activity as an Intrinsic Property of an Antibody

Stringent criteria were applied to define oxidoreductase activity as an intrinsic property of IgGs: the binding of antibodies to an IgG-affinity sorbent, the demonstration of the homogeneity of purified IgGs via electrophoresis in a polyacrylamide gel, and a match between the activity profile and the protein profile after gel filtration chromatography under acidic conditions (pH shock analysis). The list of these criteria includes the most convincing and widely applicable tests that have been used by independent research groups over the past 30 years. Below, we present the results of these experiments in more detail.

The specific interaction of the protein G Sepharose with the Fc fragment of IgG and the isolation of such fractions during affinity chromatography (followed by dialysis) allowed us to obtain IgG without contaminants.

**Figure 1:**
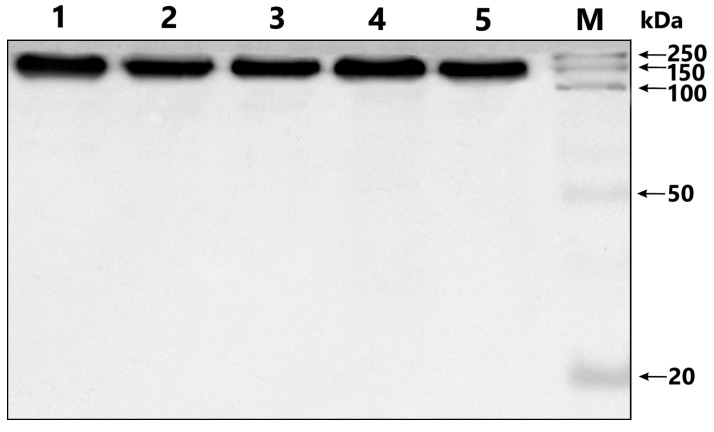
Analysis of homogeneity of IgG samples after SDS-PAGE in a 4–18% gradient gel and Coomassie G250 staining. Lanes 1–5: IgGs from different schizophrenia patients; M: protein molecular-weight markers.

The homogeneity of the 150 kDa IgG was confirmed by 4–18% SDS-PAGE (Figure 1).

The assays of the SOD and catalase activities of the IgGs from the blood serum showed that the activities remained after gel filtration under acid shock only in the fractions corresponding to the optical density profile of the antibodies and were not detectable in the other fractions.

**Figure 2:**
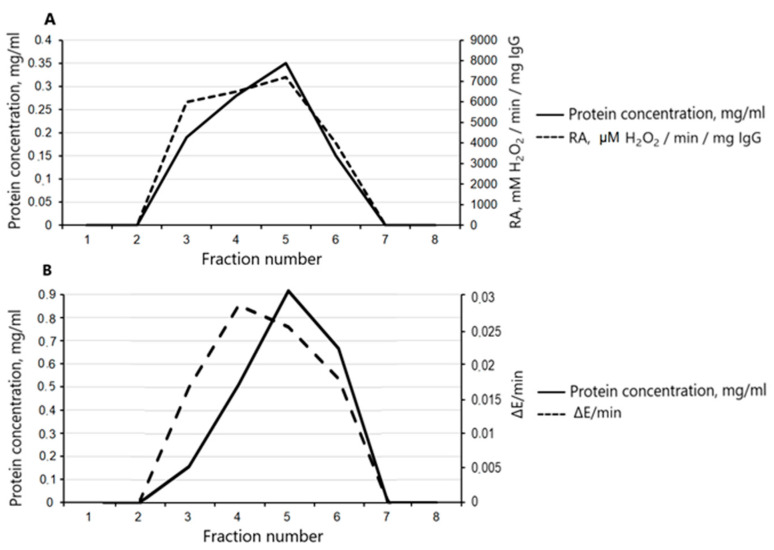
(**A**). The protein elution profile of gel filtration of IgGs under pH shock conditions (solid curve) and the change in relative activity (RA) of the IgG during the degradation of H_2_O_2_ in the presence or absence of IgG having catalase activity in the obtained fractions (dashed curve). (**B**). The protein elution profile of gel filtration of IgGs under pH shock conditions (solid curve) and the change in optical density of the solution per minute (∆E/min) in the presence or absence of IgGs having SOD activity in the obtained fractions (dashed curve).

Figure 2 presents an overlay of the absorbance profile of the eluted protein (IgG) and the levels of both the SOD and catalase activities for each individual fraction obtained during the gel filtration. The proof that the activity belongs to IgGs is the match between the SOD or catalase activity maximum and the top of the chromatographic peak corresponding to IgG.

Taken together, the results of the series of experiments clearly indicated that homogeneous individual polyclonal immunoglobulins with catalytic properties were obtained successfully.

### 3.2. Results of the Cell Experiment

The incubation of the SH-SY5Y cell line for 24 h with IgG isolated from the blood of the healthy individuals or the patients with schizophrenia was performed on the CellInsight CX7 HCS platform (Thermo Fisher Scientific, USA). For all the immunoglobulin samples, the catalase and SOD activities were determined beforehand.

We also conducted an experiment reproducing oxidative stress by adding a high concentration of TBH to the culture medium. The culture medium without this chemical served as a control. By means of the CellInsight CX7 HCS platform, the dead cells were counted at the end of the experiment.

The levels of the catalase and SOD activities of the IgG from the patients in the acute phase of schizophrenia and the healthy individuals are presented in Table 2. According to the results of the experiment, the catalase activity of the IgGs from the patients in the acute phase of schizophrenia is three times lower as compared to the healthy individuals (*p* = 0.02). The SOD activity of the IgGs from the patients was statistically significantly higher than that from the healthy people (*p* = 0.02; Table 2).

At the same time, between these two oxidoreductase activities, there was a significant strong negative correlation, r = −0.784, consistently with canonical functions of the enzymes in question: catalase and SOD.

**Figure 3:**
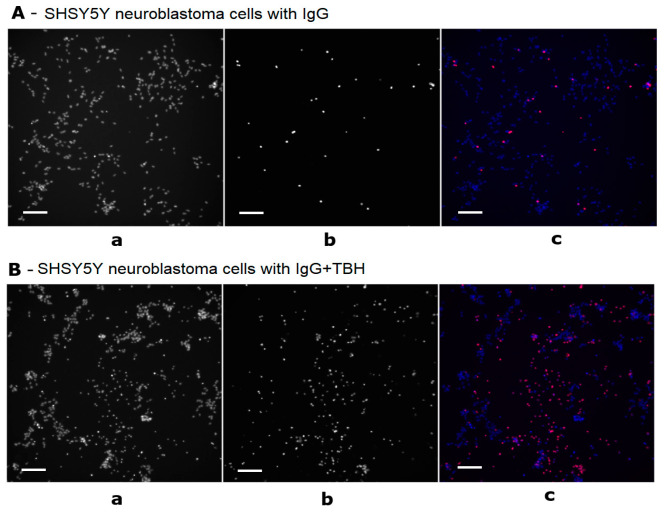
Registration of cells on the CX7 platform. (**A**): SH-SY5Y cells after 24 h of incubation with IgG from schizophrenic patients; (**B**): SH-SY5Y cells after 24 h of incubation under conditions of induced oxidative stress (TBH) along with IgG from schizophrenic patients. (**a**) Cells stained with the Hoechst fluorescent dye; (**b**) cells stained with fluorescent dye propidium iodide; (**c**) cells stained with Hoechst + propidium iodide. Scale bars: 100 μm.

The proportion of the dead cells among the SH-SY5Y cells after incubation with the IgG having a proven catalytic activity was calculated from the proportion of the nuclei stained with the fluorescent dye propidium iodide (stains the nuclei of the dead cells) among all the nuclei stained with Hoechst (Figure 3).

**Figure 4:**
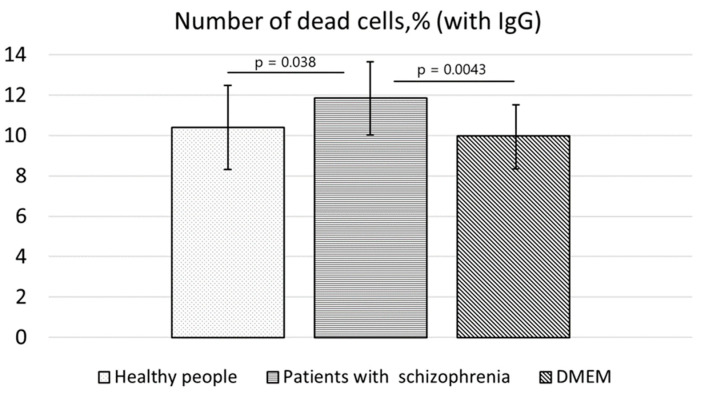
Percentages of dead SH-SY5Y cells after 24 h of incubation with IgG from schizophrenic patients or from healthy individuals or without antibodies (the culture medium alone).

After the experiment, it turned out that the immunoglobulins G from the serum of the patients in the acute phase of schizophrenia had a significant cytotoxic effect on the SH-SY5Y cell line after 24 h of incubation. A statistically significant increase in the number of dead SH-SY5Y cells was found when the antibodies from the patients were added to the culture medium, in comparison with the antibodies from the healthy individuals (*p* = 0.038); in the comparison to the incubation without antibodies, this effect was even more pronounced (*p* = 0.0043). No influence of the serum IgG from the healthy people on cell death was detectable: the number of dead cells in this case did not differ from that in the control (culture medium alone; *p* = 0.33; Figure 4).

We also tested the impact of induced oxidative stress on cell survival under the above conditions. TBH was utilized as an inducer of cell death to model oxidative stress. Preincubation with TBH resulted in a fivefold increase in the percentage of dead cells. Next, after supplementation of the medium with IgG from the schizophrenia patients, the cytotoxic effect of TBH on the SH-SY5Y cell line was still present. Moreover, the cytotoxic effect of the coincubation of the cultured cells with TBH and IgG from the patients with schizophrenia was statistically significantly much more pronounced. The increase in the percentage of dead SH-SY5Y cells under the influence of antibodies from the patients with schizophrenia was highly significant in comparison with the culture medium alone (*p* = 0.00012), and the difference from the healthy donor control was even greater (*p* = 0.000003).

**Figure 5:**
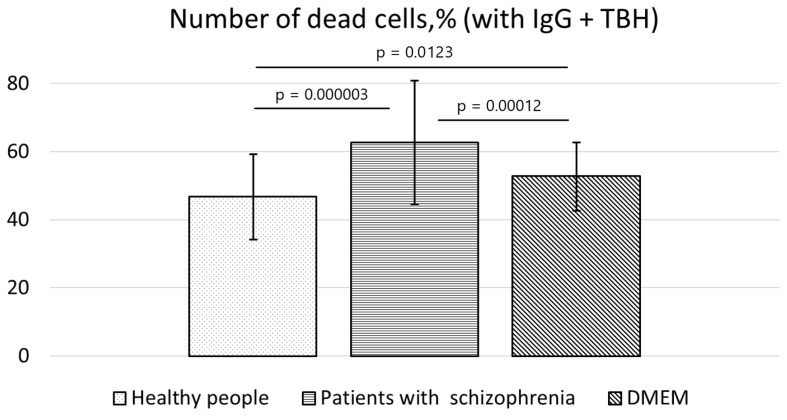
Percentages of dead SH-SY5Y cells after 24 h of induced oxidative stress (TBH) combined with incubation with IgG from schizophrenia patients or from healthy individuals or without antibodies (the culture medium alone).

Moreover, a cytoprotective property was found in the IgG samples from the healthy individuals. The number of dead SH-SY5Y cells was significantly lower in comparison with the culture medium alone (*p* = 0.0123; Figure 5).

In addition, in the group of patients with schizophrenia, a significant moderate positive correlation was found between the percentage of dead cells and the SOD activity of the patients’ IgG samples (r = 0.439) as well as a significant weak correlation between the percentage of cells showing oxidative stress-induced death and the level of catalase activity (r = 0.355).

## 4. Discussion

In schizophrenia, there is evidence of immunological dysregulation [29,30] and the activation of humoral immunity [15], along with generalized oxidative stress [12] and a local decrease in the activity of antioxidant systems [31,32]. In this regard, the experimental findings presented in this work make a substantial contribution to the understanding of the mechanisms of action of abzymes possessing oxidoreductase properties in the human body and to explaining several components of schizophrenia pathogenesis associated with oxidative stress. Our SOD assay revealed that the activity of the serum IgG from the patients with schizophrenia is significantly higher as compared to healthy people, while the catalase activity in the same pool of immunoglobulins G is significantly lower relative to healthy individuals. These opposite changes in the two oxidoreductase activities were confirmed by a significant moderate negative correlation (r = −0.784).

In the next experiment, the aim was to examine the effect of IgGs having oxidoreductase activities from schizophrenia patients or healthy individuals on cell death. We assumed that the neuroblastoma cell line was the most suitable model for the first stage of our experimental study. Human neuroblastoma cell line SH-SY5Y is the most popular in vitro model in neuropsychiatric research [33,34]. SH-SY5Y cells are widely used either as undifferentiated cells or as cells differentiated into a neuron-like lineage. There are receptors for dopamine, GABA, acetylcholine, and glutamate on the surface of SH-SY5Y cells [35]. For example, the mechanisms of action of olanzapine and haloperidol on the serum withdrawal apoptosis of SH-SY5Y cells have been investigated by N.R. Kim [36]. Accordingly, in our experiment, we incubated SH-SY5Y cells with immunoglobulins G (from patients or healthy individuals) possessing significantly different oxidoreductase activities. Our results uncovered the effects having different directions depending on the levels of catalase and the SOD activities of the IgGs.

During the 24 h of incubation in a culture plate with SH-SY5Y cells, abzymes having high SOD activity could create a sufficient pool of hydrogen peroxide not completely neutralized by the low catalase activity. At micromolar concentrations, hydrogen peroxide, along with other ROS, triggers oxidative damage to proteins, nucleic acids, and lipids, thus leading to the activation of proteolytic enzymes and the disruption of the membrane structure, inevitably causing cell death [37]. We can theorize that it is the accumulation of hydrogen peroxide in the SH-SY5Y cell culture medium that can explain the observed cytotoxic effect. After a prolonged 24 h incubation of the cell line with serum immunoglobulins G from the patients in the acute phase of schizophrenia, a significant cytotoxic effect was documented. The number of dead SH-SY5Y cells when the samples of the patients’ antibodies were added to the culture medium statistically significantly exceeded this parameter in the group of healthy individuals and in the control group, consisting of cultured cells without the addition of antibodies. This finding was confirmed by the significant positive correlation between the average magnitude of the patients’ IgG SOD activity and the percentage of dead SH-SY5Y cells (r = 0.439).

Furthermore, as already stated above, it is possible that the oxidoreductase activities of class G immunoglobulins are activated by the mechanism of substrate induction, as in classic enzymes [23,24]. This notion is confirmed in the current work. Under our conditions of induced oxidative stress, the cytotoxic effect on the SH-SY5Y cell line was much more pronounced for IgG from the patients with schizophrenia (as compared to the healthy controls), once again corroborating the major role of ROS in the regulation of cell death. The clinical relevance of this topic is constantly supported by published articles [38].

In addition, in our experiments, a very interesting phenomenon was revealed. Under the induced oxidative stress, a statistically significant cytoprotective effect of IgG from the healthy individuals on the SH-SY5Y cells was registered. Currently, this phenomenon is difficult to explain. A possible reason is that, possibly, the high catalase activity of the IgG from the healthy individuals eliminates the excess hydrogen peroxide from the culture medium, which is clearly the cause of the cytotoxic effects. We believe that a small amount of hydrogen peroxide is formed spontaneously (without the induction) in 24 h even in the DMEM alone. This supposition needs further experiments to be confirmed. Be that as it may, lately, research articles on protective properties of abzymes have begun to appear. In a 2020 paper, some authors reported the first identified antibodies with sialidase activity. These antibodies are, for example, desialyze glycoproteins, gangliosides, and red blood cells. Desialylation of dying cells may facilitate the clearance of apoptotic cells [39].

Our work has several limitations. In this project, we used polyclonal antibodies (IgG) representing a mixture of immunoglobulins with different catalytic activities in one sample. Separating natural catalytic-antibody clones from antigen-binding antibodies can be challenging (due to their similar properties and structure). The current methods for separating a mixture of abzymes depending on the structure of their catalytic site make it impossible to subsequently evaluate their activity. Our findings are the first results on the cytotoxic properties of IgG having an oxidoreductase activity. Above, we tried to explain the results, and the difficult limitation will be resolved in our next study. Furthermore, detailed data on long-term antipsychotic therapy prior to hospitalization were not available, and this fact limits the interpretation of a possible confounding influence of the pharmacotherapy.

## 5. Conclusions

During the 24 h incubation of SH-SY5Y cells with IgG abzymes from patients with schizophrenia, a significant cytotoxic effect of these abzymes was detected, which correlated with high SOD activity. Under induced oxidative stress, the cytotoxic effect of these antibodies from the patients with schizophrenia on the SH-SY5Y cell line was more pronounced, whereas for the abzymes from the healthy individuals a cytoprotective effect on the cultured cells was observed, in combination with high catalase activity. Thus, the detected influence on cell viability depends on the catalytic properties of the abzymes.

## Figures and Tables

**Table 1:** Patients’ and healthy control subjects’ characteristics.

**Healthy Control Subjects**	n = 18
Age (years)	
Median and interquartile range	44.00 [35.00; 49.00]
Male/female ratio	7/11
**Patients with Schizophrenia**	n = 16
Age (years)	
Median and interquartile range	40.50 [38.00; 45.50]
Male/female ratio	11/7
Duration of disease (years)	
Median and interquartile range	19.00 [17.00; 22.00]

**Table 2:** Catalase and SOD activities of IgGs from patients with acute schizophrenia and healthy controls.

	Healthy Controls	Patients with Schizophrenia	*p*-Value of Difference
Catalase activity of IgGs, (mM H_2_O_2_/min/mg IgG)	598.13 ± 93.38	183.07 ± 10.06	0.02
SOD activity of IgGs (μM diformazan/min/mg of protein)	182.13 ± 32.81	207.01 ± 40.91	0.02

## Data Availability

The data presented in this study are available on request from the corresponding author.
